# Preoperative Extracorporeal Membrane Oxygenation (ECMO) Cannulation in Inferior Vena Cava Tumor Thrombus: A Case Report

**DOI:** 10.7759/cureus.78254

**Published:** 2025-01-30

**Authors:** Diana Sousa, Ana T Magalhães, Diana Fonseca, André Braga

**Affiliations:** 1 Anesthesiology, São João University Hospital Center, Porto, PRT

**Keywords:** cannulation, ecmo, hemodynamic instability, inferior vena cava, kidney, obstructive shock, pulmonary embolism, radical nephrectomy, renal cell carcinoma, tumor thrombus

## Abstract

Intravascular tumor thrombus can occur in different tumor types, but it is most common in renal cell carcinoma (RCC). This can extend into the renal vein and inferior vena cava (IVC), increasing the risk of pulmonary embolism. In cases of massive pulmonary embolism, the use of venoarterial extracorporeal membrane oxygenation (ECMO) is lifesaving, allowing time for hemodynamic stability and definitive treatment.

This case involves a 51-year-old male patient who was scheduled for elective right radical nephrectomy, ipsilateral adrenalectomy, and thrombectomy due to a large RCC. Preoperative abdominopelvic magnetic resonance imaging revealed a tumor thrombus in the right renal vein extending into the IVC. Given the high embolic risk associated with hemodynamic instability, the multidisciplinary team decided to perform preoperative venoarterial ECMO cannulation with vascular access sheaths. During the intraoperative period, the patient experienced hemodynamic instability due to hemorrhagic shock. However, a transesophageal echocardiogram (TEE) demonstrated preserved biventricular function and no evidence of right ventricular dilation, leading to withholding initiation of ECMO. Postoperative thoracoabdominal computed axial tomography scan showed segmental pulmonary thromboembolism of the right lower lobe of the lung, yet the patient remained hemodynamically stable.

In our case, during the preoperative period, we focused on optimizing the patient’s clinical condition and proceeded with ECMO cannulation using introducer sheaths. This case underscores the critical role of a multidisciplinary approach in preoperative assessment and highlights the importance of anticipating potential perioperative complications.

## Introduction

Renal cell carcinoma (RCC) constitutes approximately 3% of all cancers [1]. It is the predominant type of kidney cancer, accounting for 80%-85% of all primary renal neoplasms [2-4].

Intravascular tumor thrombus, although relatively uncommon, can occur in different tumor types. However, it is most frequently observed in RCC [1]. Venous migration (VM) and the formation of venous tumor thrombus (VTT) may also occur, particularly when there is extension into the renal vein and inferior vena cava (IVC), with a prevalence ranging from 4% to 10% [3,4]. Patients with such conditions face an elevated risk of pulmonary embolism, leading to increased morbidity, mortality, and hospitalization costs [1,5]. The presence of VM and VTT formation are distinctive features of RCC, and their occurrence, especially when accompanied by metastases, represents a critical adverse prognostic factor [6].

Currently, the standard treatment for RCC with VTT involves radical nephrectomy with thrombectomy [7]. A multidisciplinary approach, which includes urologists, oncologists, radiologists, anesthesiologists, and cardiothoracic and vascular surgeons, ensures comprehensive preoperative evaluation, optimal surgical planning, and coordinated perioperative care. This recommended approach not only improves safety but also reduces morbidity and mortality, ultimately enhancing overall outcomes for patients with RCC with VTT [8]. These patients may require a venovenous bypass or cardiopulmonary bypass, with or without circulatory arrest [9].

Extracorporeal membrane oxygenation (ECMO) is a cardiopulmonary bypass technique developed to provide temporary support for heart or lung function, ensuring oxygenation and organ perfusion [10,11]. The venovenous ECMO technique is commonly employed for severe and refractory adult respiratory failure, while venoarterial ECMO is used to support cardiorespiratory failure [12]. Currently, ECMO finds widespread use, even in the operating room [10]. In cases of massive pulmonary embolism, venoarterial ECMO can be a lifesaving intervention, allowing time for hemodynamic stability and definitive treatment [10,13].

In this clinical case, we describe a 51-year-old male patient with RCC and tumor thrombus that invades the renal vein and extends into the IVC. The decision was made to perform preoperative ECMO cannulation with the placement of introducer sheaths. Our experience highlights the importance of a multidisciplinary approach in managing this complex surgical situation, ultimately leading to improved outcomes. This case report was previously presented as a meeting poster at the 18th World Congress of Anesthesiologists on March 3-7, 2024.

## Case presentation

A 51-year-old male patient with an American Society of Anesthesiologists (ASA) III physical status classification, presented with a history of systemic arterial hypertension and right RCC, was proposed for elective right radical nephrectomy, ipsilateral adrenalectomy, and thrombectomy. 

**Figure 1:**
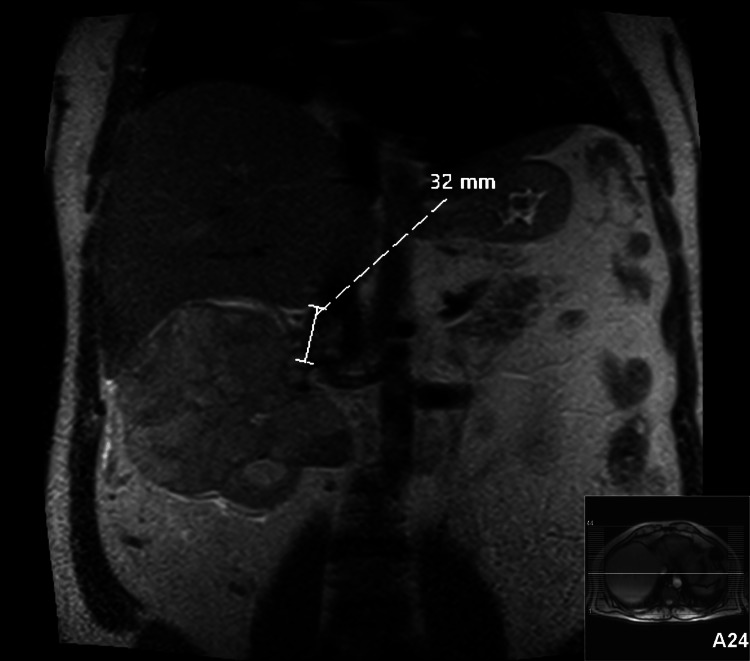
Abdominopelvic magnetic resonance imaging: a 32 mm tumor thrombus within the right renal vein, extending into the inferior vena cava

The preoperative analytical study revealed anemia with a hemoglobin level of 11.3 g/dl and a slight elevation of serum creatinine (measuring 1.42 mg/dl). No other significant analytical changes were detected. Both the electrocardiogram and echocardiogram results were unremarkable. However, the abdominopelvic magnetic resonance imaging demonstrated a 32 mm tumor thrombus within the right renal vein, extending into the IVC at level II (Figure 1).

Attending to the complexity of the case, a multidisciplinary approach was employed, involving two anesthesiologists, one radiologist, three urologic surgeons, two vascular surgeons, and three members of the ECMO team. Due to the location of the tumor thrombus in the IVC, the patient was at high risk for embolic events, which could lead to significant hemodynamic instability. In light of this, the potential need for mechanical circulatory and respiratory support during the intraoperative period was carefully considered. Taking into account this elevated risk associated with the patient's age and minimal comorbidities of systemic hypertension, the decision was made to perform preoperative venoarterial ECMO cannulation with vascular access sheaths. On the day before surgery, the ECMO team successfully inserted introducer sheaths into the right jugular vein (9 Fr) and in the left femoral artery (6 Fr) under ultrasound guidance, with no complications reported. This preemptive approach was chosen for its minimally invasive nature while ensuring that rapid and definitive ECMO cannulation could be readily achieved if required during the procedure.

Due to the thrombus in the IVC, the patient was anticoagulated with a therapeutic dose of enoxaparin, 60 mg administered twice daily. Before cannulation, the enoxaparin was suspended, and the activated partial thromboplastin time, prothrombin time, and platelet count were confirmed to be within the normal range, following the European Society of Anaesthesiology and Intensive Care (ESAIC) and the European Society of Regional Anaesthesia (ESRA) guidelines. Upon admission for the procedure, the patient presented with a blood pressure of 138/81 mmHg, a heart rate of 73 bpm, a respiratory rate of 14 breaths per minute, a tympanic temperature of 36.2ºC, and an oxygen saturation of 97% in room air.

At the time of surgery, the patient underwent general anesthesia. During the intraoperative period, the patient experienced hemodynamic instability due to hemorrhagic shock, requiring a transfusion of 4 units of crossmatched A Rh-positive red blood cells, 5 liters of polyelectrolytic crystalloids, and vasopressor support via noradrenaline infusion (maximum dose of 0.56 $\mu$g/kg/min). An intraoperative transesophageal echocardiogram (TEE) was performed, and it demonstrated preserved biventricular function and no evidence of right ventricular dilation, tricuspid regurgitation, or elevated pulmonary artery pressure. Consequently, this led to the decision not to initiate ECMO. Hemoglobin was 9.6 g/dl in the final arterial gas blood analysis. Following the procedure, the patient was transferred to the Intensive Care Unit under mechanical ventilation. Later that day, the noradrenaline perfusion was discontinued, and the patient was extubated, uneventfully.

**Figure 2:**
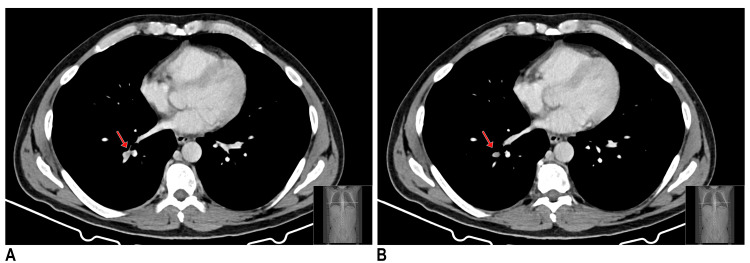
Thoracoabdominal computed tomography axial scan: segmental pulmonary thromboembolism of the right lower lobe of the lung A: segment 1; B: segment 2

In the postoperative period, a thoracoabdominal computed tomography (CT) axial scan was conducted for surgical reassessment. In this exam, a segmental pulmonary thromboembolism of the right lower lobe of the lung was identified (Figure 2).

Anticoagulation therapy with enoxaparin, 60 mg twice daily, was promptly initiated to ensure effective management. The patient remained asymptomatic and hemodynamically stable and did not require supplemental oxygen therapy. ECMO support was deemed unnecessary.

## Discussion

Intravascular tumor thrombus is defined as tumor extension into a vessel [3]. Its presence changes the stage, prognosis, and treatment. While it can be seen in a range of tumors, including Wilms tumor, adrenal cortical carcinoma, and hepatocellular carcinoma, it is most frequently associated with RCC [3,5]. In RCC, VTT can extend to both the renal vein and the IVC, thereby increasing the risk of pulmonary embolism [5]. According to the American Joint Committee on Cancer (AJCC) staging system, several studies have shown improved survival outcomes in RCC patients with isolated renal vein involvement compared to those with IVC thrombus [6]. An alternative and more detailed classification system for tumor thrombus is the Mayo Clinic classification, which aids in surgical planning (Table 1) [14].

**Table 1:** Mayo Clinic classification of venous tumor thrombus Adapted/reproduced from Blute et al. [14], with permission

Thrombus level	Description
0	Thrombus extending to the renal vein
I	Thrombus extending into the inferior vena cava to no more than 2 cm above the renal vein
II	Thrombus extending into the inferior vena cava to more than 2 cm above the renal vein but not to the hepatic vein
III	Thrombus extending into the inferior vena cava to above the hepatic vein but not to the diaphragm
IV	Thrombus extending into the supradiaphragmatic inferior vena cava or right atrium

However, the use of VTT as a reliable prognostic predictor remains controversial [6]. While advancements in surgical techniques and perioperative management have been made, the overall survival of these patients remains relatively poor, with a five-year overall survival rate of around 39% [7].

Radical nephrectomy with thrombectomy remains the standard treatment for RCC with VTT, which was the surgery proposed in this case. However, it may be responsible for physiological changes that increase morbidity and mortality, depending on the patient’s age and overall health status [15]. Anesthesia plays an essential role in the management of this condition. To ensure the quality and safety of both anesthesia and surgery, it is essential to develop a precise individualized plan based on the extent of the disease, a thorough understanding of the patient’s clinical characteristics, and an awareness of available treatment options [8,15]. The management of this condition is increasingly reliant on a multidisciplinary approach. Hospitals that have perioperative care teams are better at identifying and mitigating perioperative complications [16]. So, in our specific case, given the complexity of the surgery and associated risks, we adopted a multidisciplinary approach throughout the perioperative period.

The multidisciplinary management of our patient included anesthesiologists, radiologists, surgeons, and ECMO teams. According to Master et al., a comprehensive multidisciplinary approach, involving urologists, oncologists, radiologists, anesthesiologists, and vascular surgeons, significantly enhances the overall outcomes of the patients with RCC with VTT [8].

In recent years, the use of ECMO has increased in perioperative care. The primary indications for ECMO include clinical conditions that require short-term respiratory and/or circulatory support when conventional methods fail to maintain life. ECMO serves the dual purpose of allowing time for hemodynamic stability and facilitating definitive treatment. It can be employed for preoperative stabilization or as emergency support during intraoperative and postoperative complications, such as obstructive and cardiogenic shock [11]. According to these assumptions and the significant embolic risk associated with hemodynamic instability in our patient, we discussed preoperatively the potential need for mechanical circulatory support during intraoperative time. Consequently, a decision was made to perform preoperative ECMO cannulation with the placement of introducer sheaths. 

Although ECMO has great advantages, cannulation is associated with important complications. These can be classified into mechanical-related or patient-related complications. Mechanical complications include failure of the oxygenator, rupture of the circulation pipeline, failure of the pump or pressure sensor, and displacement of catheter position. Patient-related complications include bleeding, hemolysis, nervous system complications, organ failure, infection, and metabolic disorders, including electrolyte imbalances, acid-base disorders, and hyperglycemia [17]. In our case, ECMO cannulation was successfully performed without any recorded complications.

In the surgical management of level II-IV tumor thrombi involving the IVC, intraoperative TEE plays a crucial role [1]. It allows real-time monitoring with the assessment of cardiac function and early detection of thrombus embolization during IVC manipulation [9]. The overall incidence of intraoperative embolization is approximately 1.5%, with the risk being higher for higher-level thrombi. This complication carries a significant mortality rate of 75% [9]. Given the significant mortality risk associated with intraoperative embolization, vigilant monitoring and prompt intervention are essential to optimize patient safety and outcomes. Therefore, in situations of sudden hemodynamic instability, a TEE should be done to assess the presence of thrombus embolization [9]. In the present clinical case, TEE was used as the main method of hemodynamic monitoring. During the episode of hemodynamic instability, TEE revealed preserved biventricular function with no evidence of right ventricular dilation, tricuspid regurgitation, or elevated pulmonary artery pressure. This allowed us to assess not only the cardiac function but also the underlying etiology of the patient’s shock. This intraoperative assessment provided valuable information that assisted the multidisciplinary team in excluding the possibility of a massive pulmonary embolism, thereby supporting the decision to withhold ECMO. Although intraoperative massive pulmonary embolism was ruled out, the postoperative thoracoabdominal CT identified segmental pulmonary thromboembolism in the right lower lobe.

## Conclusions

In summary, cases of RCC with tumor thrombus invading the IVC present unique challenges for perioperative management, which arise from the potential need for complex surgical intervention. This case underscores the critical importance of a thorough preoperative assessment and the anticipation of potential critical events. A perioperative multidisciplinary approach, integrating anesthesiology, radiology, intensive care medicine, and surgical teams, enables the use of advanced techniques like ECMO, thereby contributing to enhance the overall outcome.
